# Thymoquinone is a natural antibiofilm and pathogenicity attenuating agent in *Pseudomonas aeruginosa*

**DOI:** 10.3389/fcimb.2024.1382289

**Published:** 2024-04-04

**Authors:** Mohammed W. Al-Rabia, Hani Z. Asfour, Nabil A. Alhakamy, Wesam H. Abdulaal, Tarek S. Ibrahim, Hisham A. Abbas, Ibrahim M. Salem, Wael A. H. Hegazy, Shaimaa I. Nazeih

**Affiliations:** ^1^ Department of Clinical Microbiology and Immunology, Faculty of Medicine, King Abdulaziz University, Jeddah, Saudi Arabia; ^2^ Department of Pharmaceutics, Faculty of Pharmacy, King Abdulaziz University, Jeddah, Saudi Arabia; ^3^ Center of Excellence for Drug Research and Pharmaceutical Industries, King Abdulaziz University, Jeddah, Saudi Arabia; ^4^ Mohamed Saeed Tamer Chair for Pharmaceutical Industries, King Abdulaziz University, Jeddah, Saudi Arabia; ^5^ Department of Biochemistry, Faculty of Science, Cancer and Mutagenesis Unit, King Fahd Medical Research Center, King Abdulaziz University, Jeddah, Saudi Arabia; ^6^ Department of Pharmaceutical Chemistry, Faculty of Pharmacy, King Abdulaziz University, Jeddah, Saudi Arabia; ^7^ Department of Microbiology and Immunology, Faculty of Pharmacy, Zagazig University, Zagazig, Egypt; ^8^ Department of Pharmaceutical Chemistry, Faculty of Pharmacy, Sphinx University, Assiut, Egypt; ^9^ Pharmacy Program, Department of Pharmaceutical Sciences, Oman College of Health Sciences, Muscat, Oman

**Keywords:** thymoquinone, antibiofilm, *Pseudomonas aeruginosa*, antibiotic resistance, virulence inhibition, healthcare

## Abstract

*Pseudomonas aeruginosa* belongs to the critical pathogens that represent a global public health problem due to their high rate of resistance as listed by WHO. *P. aeruginosa* can result in many nosocomial infections especially in individuals with compromised immune systems. Attenuating virulence factors by interference with quorum sensing (QS) systems is a promising approach to treat *P. aeruginosa*-resistant infections. Thymoquinone is a natural compound isolated from *Nigella sativa* (black seed) essential oil. In this study, the minimum inhibitory concentration of thymoquinone was detected followed by investigating the antibiofilm and antivirulence activities of the subinhibitory concentration of thymoquinone against *P. aeruginosa* PAO1. The effect of thymoquinone on the expression of QS genes was assessed by quantitative real-time PCR, and the protective effect of thymoquinone against the pathogenesis of PAO1 in mice was detected by the mouse survival test. Thymoquinone significantly inhibited biofilm, pyocyanin, protease activity, and swarming motility. At the molecular level, thymoquinone markedly downregulated QS genes *lasI*, l*asR*, *rhlI*, and *rhlR*. Moreover, thymoquinone could protect mice from the pathologic effects of *P. aeruginosa* increasing mouse survival from 20% to 100%. In conclusion, thymoquinone is a promising natural agent that can be used as an adjunct therapeutic agent with antibiotics to attenuate the pathogenicity of *P. aeruginosa*.

## Introduction

*Pseudomonas aeruginosa* is a common opportunistic and nosocomial pathogen that impacts individuals with compromised immune systems (Gellatly and Hancock, 2013), such as patients suffering from chronic obstructive pulmonary disease (COPD), healthcare-associated pneumonia, or cystic fibrosis (Bjarnsholt et al., 2009; Garcia-Nuñez et al., 2017; Wunderink and Waterer, 2017). *P. aeruginosa* is listed among the “critical” category of the World Health Organization’s (WHO) priority bacterial pathogens. The bacteria in such category are highly resistant to antibiotics, so it is necessary to develop new strategies to treat infections caused by these resistant bacteria (Bjarnsholt et al., 2009; Almalki et al., 2022c; Nazeih et al., 2023). *P. aeruginosa* pathogenicity is mediated by a large group of both virulence factors and antibiotic resistance determinants. Thanks to this group of virulence and resistance, *P. aeruginosa* shows remarkable metabolic flexibility and adaptability to hostile conditions, including the host immune response (Tacconelli et al., 2018; Botelho et al., 2019). The treatment of *P. aeruginosa* infections is challenging due to its multiple drug tolerance mechanisms, either intrinsic, acquired, or adaptive (Botelho et al., 2019; Thabit et al., 2022a).

*P. aeruginosa* is known for its ability to form robust and resilient biofilms that contributes to its adaptability and persistence in various environments, including medical settings and host tissues (Khayat et al., 2022c; Alotaibi et al., 2023). Bacterial biofilms are structured communities of microorganisms that adhere to surfaces and encase themselves in a self-produced extracellular matrix composed of polysaccharides, proteins, and DNA (Hegazy et al., 2022; Lila et al., 2023). The formation of biofilms has significant implications for both the virulence of bacteria and their resistance to antibiotics. In terms of virulence, the biofilm matrix constitutes a protective barrier that hinders the access of immune cells to the bacteria within the biofilm and help the biofilm cells to resist the host immunity (Elfaky et al., 2023; Rajab and Hegazy, 2023). The complex architecture of biofilms allows bacteria to establish persistent infections (Hegazy and Abbas, 2017; Cavalu et al., 2022). Furthermore, bacteria within biofilms exhibit increased tolerance to antibiotics compared with their planktonic counterparts, thanks to the biofilm matrix barrier that prevents the penetration of antimicrobial agents and shielding bacteria from their effects (Rajab and Hegazy, 2023). Additionally, the metabolic changes and altered gene expression within biofilms contribute to antibiotic resistance by rendering bacteria dormant and less susceptible to the action of antimicrobial agents (Alandiyjany et al., 2022; Lila et al., 2023).

Among other virulence factors, biofilm formation is mainly controlled by a cell-to-cell communication system named quorum sensing (QS) that is based on sensing the number of bacterial cells by means of autoinducers or signaling molecules whose concentrations correspond to the cell numbers. When the autoinducer concentrations reach a certain threshold, they bind to their cognate receptors. This binding leads to activation of the expression of virulence genes (Almalki et al., 2022a; Koshak et al., 2024). *P. aeruginosa* utilizes N-acylated homoserine lactone (AHL) signaling molecules. There are three main QS systems: LasI-LasR, RhlI-RhlR, and PQS-MvfR. Las I in the LasI-LasR system is responsible for production of N-(3-oxododecanoyl)-L-homoserine or the C12-HSL signal molecule, whereas LasR is the receptor that binds to C12-HSL. Similarly, RhlI controls the production of the N-butyryl-L-homoserine lactone or C4-HSL autoinducer that binds to the RhlR receptor (Venturi, 2006; Khayat et al., 2022a). The third QS system is PQS-MvfR, with 2-heptyl-3-hydroxy-4(1H) quinolone as the autoinducer and MvfR as the binding receptor (Ilangovan et al., 2013). As a consequence, targeting QS is advantageous and results in attenuation of the bacterial pathogenesis without affecting the bacterial growth or stimulating the emergence of resistance (Bjarnsholt et al., 2013; Khayat et al., 2023b). The effectiveness of this approach has been validated in controlling the bacterial virulence in many studies (Agha et al., 2016; Lila et al., 2023). Optimizing the advantages of this approach can be achieved by utilizing compounds recognized by their safety as natural products (Abbas and Hegazy, 2017; Khayat et al., 2023b).

Plants produce a diverse array of secondary metabolites as a means to safeguard themselves from various pathogens present in their environment (Mohammed et al., 2021; Amin et al., 2022). *Nigella sativa* is an invaluable plant that grows naturally in northern Africa, southern Europe, and southern Asia (Qureshi et al., 2022). Thymoquinone (TQ), a primary bioactive constituent found in the essential oils of *N. sativa*, exhibits a spectrum of biological activities, including antimicrobial, antiparasitic, antiviral, anti-inflammatory, and anticancer properties (Forouzanfar et al., 2014; Darakhshan et al., 2015; Qureshi et al., 2022). Thymoquinone was selected as a potential QS inhibitor because it is a benzoquinone compound. In a previous study, it was found that the benzoquinone compound 2-tert-butyl-1,4-benzoquinone exerted a QS inhibitory activity in the reporter strain *C. violaceum* ATCC 12472 (Xu et al., 2022).

This study aims to explore the potential antibiofilm and antivirulence activities of TQ against the PAO1 strain of *P. aeruginosa*. This exploration is conducted in the context of TQ’s ability to interfere with quorum sensing (QS) mechanisms.

## Materials and methods

### Bacteria, media, and growth conditions

The *P. aeruginosa* PAO1 strain was obtained from the Department of Microbiology, Faculty of Pharmacy, Mansoura University. The media were purchased from Lab M Limited, Lancashire, United Kingdom. All used chemicals were of pharmaceutical grade. Dimethyl sulfoxide (DMSO) and thymoquinone (TQ) were bought from Sigma (St. Louis, USA).

### Determination of minimum inhibitory concentration of thymoquinone

Different twofold serial dilutions of thymoquinone were prepared in Mueller–Hinton (MH) broth (0.125 mg/ml–8 mg/ml), and these dilutions were added to the wells of the microtiter plate (100 µl aliquots). An overnight culture of PAO1 was diluted in Mueller–Hinton broth in order to prepare a suspension (1 × 10^6^ CFU/ml). The suspension was added to the wells containing the thymoquinone dilutions in 100-µl aliquots, and the microtiter plate was incubated at 37°C for 20 h, after which the minimum inhibitory concentration (MIC) was determined by detecting the least concentration of thymoquinone that inhibited visible growth (Nazeih et al., 2023). In all next experiments, ¼ MIC of thymoquinone (125 µl/ml) was used to assess its antivirulence and anti-QS potential activities.

### Detection of the bacterial growth inhibition by subinhibitory concentration of thymoquinone

To ensure that TQ had no effect on bacterial growth at the used subinhibitory concentration, the growth in the presence of TQ was assessed and compared with the growth in control untreated PAO1 (Nalca et al., 2006; Thabit et al., 2022b). Tubes of LB broth containing subinhibitory concentrations of TQ and control LB were inoculated with PAO1 from an overnight culture. The optical densities of test and control Luria–Bertani (LB) broth were measured at 600 nm after overnight incubation at 37°C and compared along with an investigation if there is any statistically significant difference.

### Assay of biofilm inhibition

To estimate the possible TQ antibiofilm activity, the modified method of Stepanovic et al. was used (Stepanović et al., 2007; Elfaky et al., 2022). PAO1 was overnight grown in Tryptone Soya Broth (TSB) at 37°C and then diluted with TSB to a final concentration of 1 × 10^6^ CFU/ml. Bacterial suspensions (0.1 ml) were added to 96-well sterile microtiter plate wells that contain 0.1-ml aliquots of TSB or TSB with ¼ MIC of TQ. After being incubated for 24 h at 37°C, the planktonic cells were sucked out and the wells were washed, dried in air followed by fixation of the biofilm cells by adding methanol (99%) for 20 min. Crystal violet (CV) (1%) was used to stain the wells, and the excess stain was washed off after 20 min. Glacial acetic acid (33%) was added to solubilize the attached CV, and the absorbance was measured at 590 nm.

### Swarming motility inhibition assay

To investigate if TQ inhibits swarming motility, swarming LB agar plates with agar (0.5%) containing either thymoquinone (¼ MIC) or control plates were used. Overnight cultures of PAO1 in tryptone broth were diluted, and 2 µl was surface inoculated on plates followed by incubation for 18 h at 37°C. The swarming zones were measured and compared (Rashid and Kornberg, 2000).

### Protease inhibition assay

The potential ability of TQ to decrease the PAO1proteolytic activity was assessed using the skim milk agar method (Vijayaraghavan and Vincent, 2013). The supernatants obtained from centrifugation of overnight cultures of PAO1 in LB broth with and without TQ at ¼ MIC were added in aliquots of 100 µl into the preformed wells into skim milk agar plates (5%). The plates were overnight incubated at 37°C to measure the formed clear zones around the wells.

### Pyocyanin inhibition assay

TQ was investigated for its inhibiting activity against the PAO1 virulent blue-green pyocyanin pigment (Das and Manefield, 2012). LB broth was used for growing PAO1 that was incubated overnight, followed by dilution to reach an OD of 0.4 at 600 nm. Another set of LB broth tubes with 1 ml each either containing TQ at ¼ MIC or without TQ was inoculated with 10 μl of the prepared suspension and incubated at 37°C for 48 h. The supernatants were separated by centrifugation, and the absorbance of pyocyanin was measured at 691 nm.

### Effect of thymoquinone on the expression of QS genes

In order to estimate the levels of QS gene expression in the presence and absence of TQ at ¼ MIC, rt-PCR was performed. RNA from PAO1 cultures either TQ-treated or untreated was extracted using GeneJET RNA Purification Kit (Thermo Scientific, USA) (Hegazy and Henaway, 2015; Askoura et al., 2022). RNA was purified and stored at −70°C until use. The expression of the QS genes was normalized to the housekeeping gene *ropD*, and the sequences of the used primers were listed in previous studies (Khayat et al., 2022b). For cDNA amplification, the SensiFAST™ SYBR^®^ Hi-ROX One-Step Kit from Bioline, UK, was used following the protocol. The StepOne Real-Time PCR system from Applied Biosystems, USA, was employed. Relative gene expression was determined using the comparative threshold cycle (ΔΔCt) method (Livak and Schmittgen, 2001).

### Mice protection test

The protective ability of TQ against the PAO1 pathogenesis was investigated using a mouse survival model (Kim et al., 2015; Khayat et al., 2023a). PAO1 overnight cultures were made in LB broth with and without TQ at ¼ MIC and diluted to a cell density of 2.5 × 10^7^ CFU/ml in phosphate-buffered saline (PBS). Three-week-old female albino mice (*Mus musculus*) were separated into five random groups of five mice each. The mice in group 1 were intraperitoneally injected with 100 µl of TQ-treated bacteria, whereas group 2 mice were injected with 100 µl of PAO1. On the other hand, the mice in group 3 were injected with DMSO, whereas groups 4 and 5 served as negative controls, where group 4 mice were injected with sterile PBS and group 5 mice were uninoculated. The mice were normally fed and kept with normal aeration at room temperature. The mice’s death was documented every day for 5 days, and the results were calculated using the log-rank test and plotted using the Kaplan–Meier method.

### *In silico* study on the binding ability of TQ to QS receptors QscR, LasR, RhlR, and PqsR

The validated molecular docking study was performed using flexible docking protocol (Salem et al., 2022) to ensure the antivirulence activity of the inspected TQ compound against different biological targets. The most stable conformers of the designated TQ and the selected reference ligands were constructed in a 3D representation fashion. The selected biological targets are *P. aeruginosa* virulence QS targets: QscR receptor (PDB entry: 3SZT) (Lintz et al., 2011), LasR-type (PDB ID: 2UV0) (Bottomley et al., 2007; Almalki et al., 2022b), RhlR receptor (PDB ID:7R3H) (Borgert et al., 2022), and PqsR receptor (PDB ID: 4JVC) (Ilangovan et al., 2013). All the targets were obtained from the Research Collaborator for Structural Bioinformatics Protein Data Bank (RCSB-PDB), where they were protonated after releasing the amino acid residue backbones and side chains, and then the target structures were subjected to a refinement protocol by automatic connect and type to correct the loosed bonds during X-ray crystallography followed by protein potential fixation where the constraints on the enzymes were gradually removed and minimized until the RMSD gradient was 0.01 kcal/mol Å. The active sites of the denoted targets in this study were detected using a radius of 10.0 Å around the inserted thymoquinone compound and the reference ligands. Conformational analysis of the investigated thymoquinone and the reference ligands was performed using the MMFF94 force field and subjected to flexible alignment adjusting the energy cutoff to 15 kcal/mol and RMSD tolerance to 0.5. A complete docking study was carried out for each compound from the scrutinized thymoquinone and the selected ligands against each target to confirm their fitting on the target active site. Correspondingly, we identified and visualized the numerous two-dimensional and three-dimensional binding interactions presented among all the investigated compounds and the adjoining amino acid residues of the active sites, where the amino acid residues (gray lines) located within a 5-Å radius distance from the bound ligand and labeled with a sequence number. Also, we envisaged three-dimensional cartoon representations of each binding domain co-crystallized with the investigated compounds and the reference ligands.

### Statistical analysis

Paired t-test was used to detect the statistical significance of the effects of TQ, and *p* values < 0.05 were considered statistically significant.

## Results

### Antibacterial activity of thymoquinone against *P. aeruginosa* PAO1

TQ inhibited the PAO1 growth at a concentration of 500 μg/ml. The concentration used in all next experiments was 125 μg/ml (1/4 MIC).

### Thymoquinone did not influence the growth of PAO1

**Figure 1:**
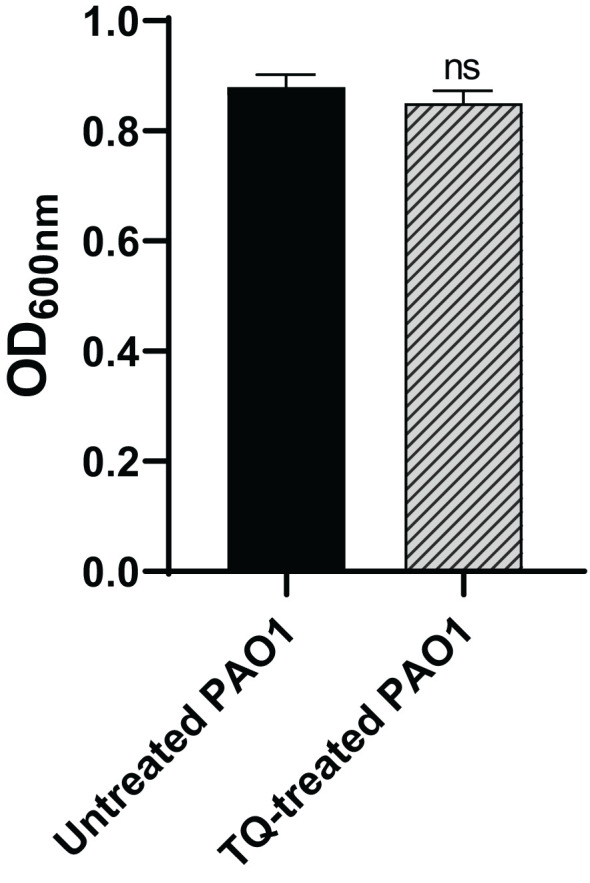
Effect of thymoquinone on PAO1 growth. No significant statistical difference was found in growth of PAO1 in the presence or absence of thymoquinone. ns, non-significant.

Before proceeding the virulence inhibition by TQ, it was necessary to ensure that the used concentration had no impact on bacterial growth. The turbidities of both treated overnight cultures of PAO1 and control cultures were compared. There was no significant difference between the turbidities in TQ treated and untreated PAO1 cultures (**Figure 1**).

### Antibiofilm activities of thymoquinone

**Figure 2:**
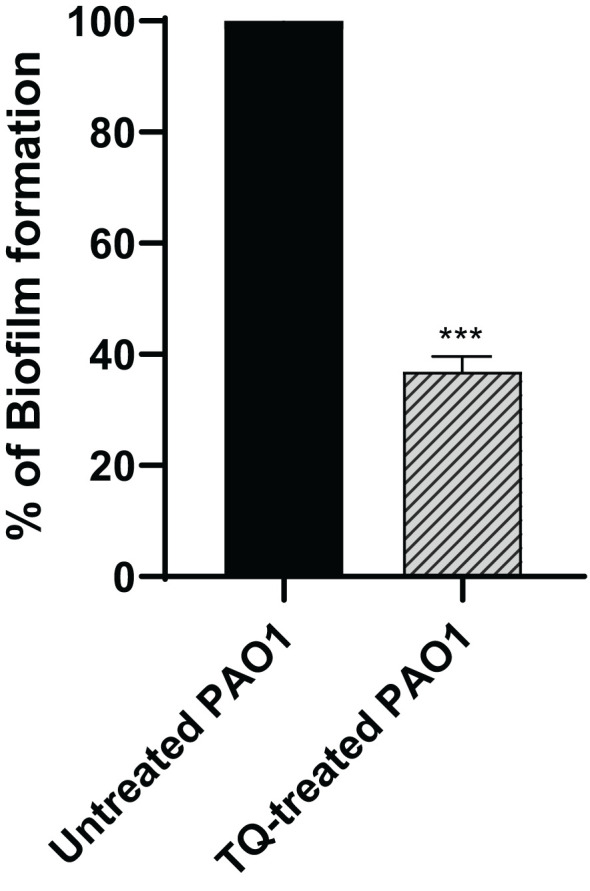
Thymoquinone antibiofilm activity in *P. aeruginosa* PAO1. Thymoquinone significantly reduced biofilm formation. The data are presented as percent change from the untreated controls. ***p < 0.001.

The crystal violet spectrophotometric method was used for evaluation of biofilm inhibition potential by TQ. The subinhibitory concentration of thymoquinone could reduce biofilm formation of PAO1 by approximately 63% (**Figure 2**).

### Proteolytic activity inhibition assay

**Figure 3:**
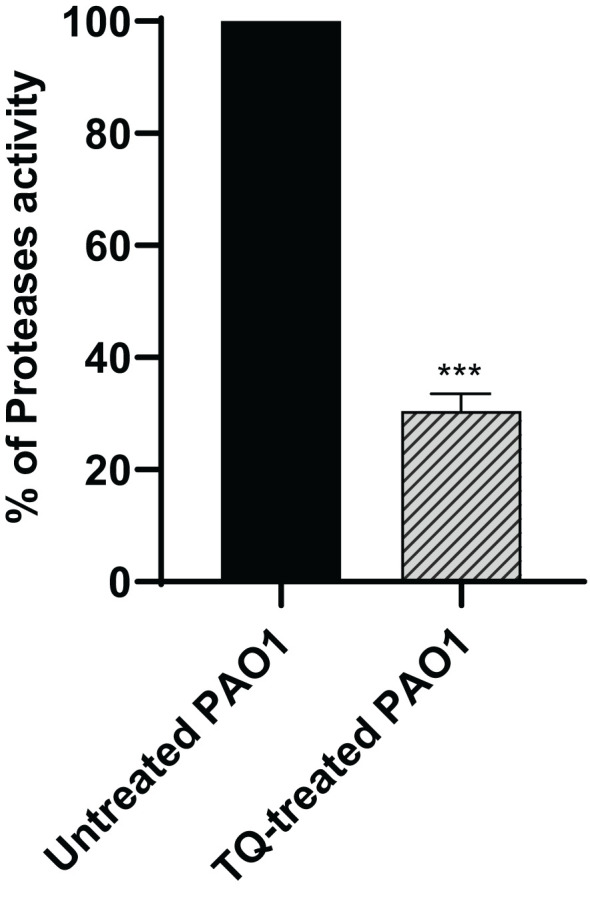
Inhibition of protease activity in *P. aeruginosa* PAO1 by thymoquinone. Thymoquinone significantly reduced protease (***p < 0.001). The data are shown as percent change from untreated controls.

The skim milk agar method was used for protease inhibition evaluation, in which the clear zones around the wells to which the supernatants of either control cultures or cultures treated with TQ at sub-MIC were added were measured and compared. TQ could significantly inhibit protease by 70% (**Figure 3**).

### Pyocyanin inhibition assay

**Figure 4:**
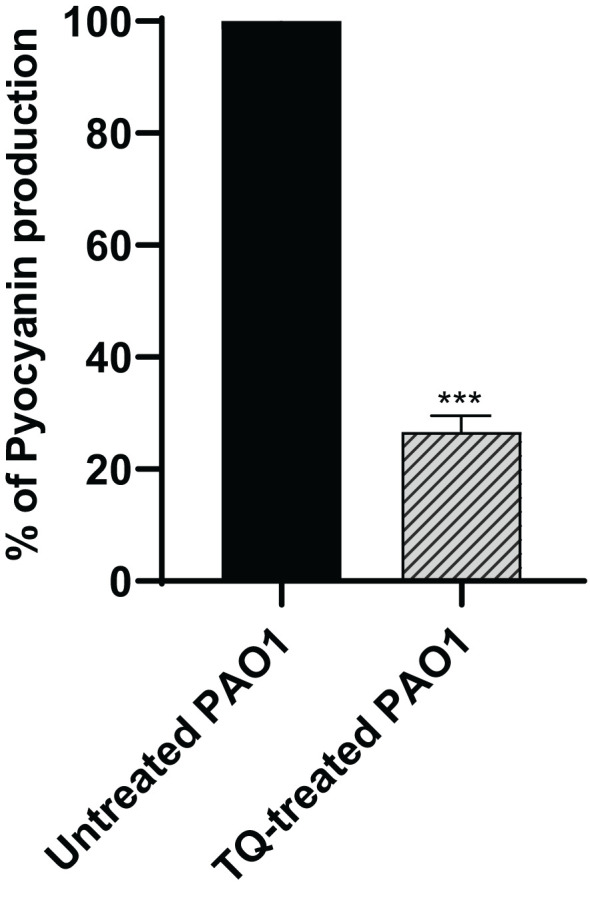
Inhibition of pyocyanin secretion by thymoquinone in *P. aeruginosa*. Significant reduction of pyocyanin secretion was found with thymoquinone (***p < 0.001). The results are shown as percent change from untreated controls.

The production of the antioxidant blue green pyocyanin pigment in *P. aeruginosa* was assayed spectrophotometrically when the bacteria were treated with TQ at sub-MIC as compared with control culture, and this revealed a significant decrease in pyocyanin by approximately 73% (**Figure 4**).

### Inhibition of swarming motility

**Figure 5:**
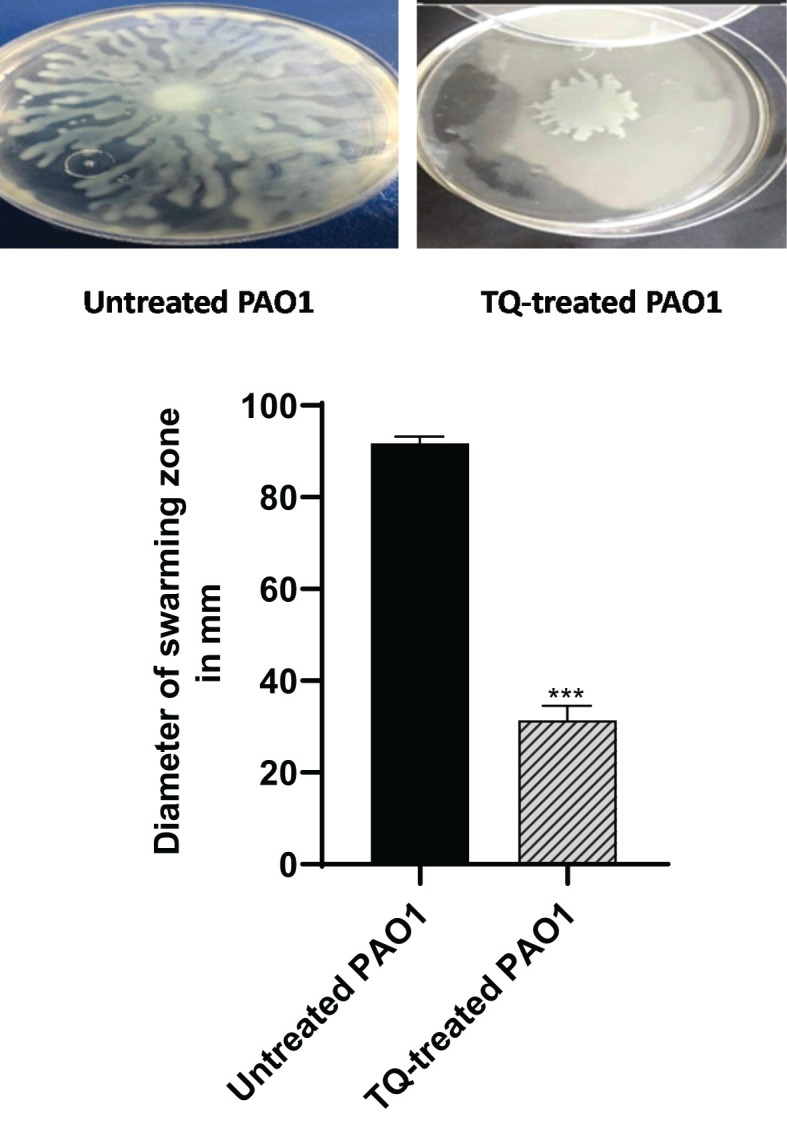
Inhibition of swarming motility of *P. aeruginosa* by thymoquinone. The swarming areas were significantly reduced by thymoquinone (***p < 0.001).

The swarming motility of PAO1 was investigated when TQ was added at sub-MIC to swarming agar as compared with control plates. TQ significantly reduced the ability of PAO1 to swarm by approximately 66% (**Figure 5**).

### Thymoquinone decreased *P. aeruginosa* capacity to induce pathogenesis *in vivo*

**Figure 6:**
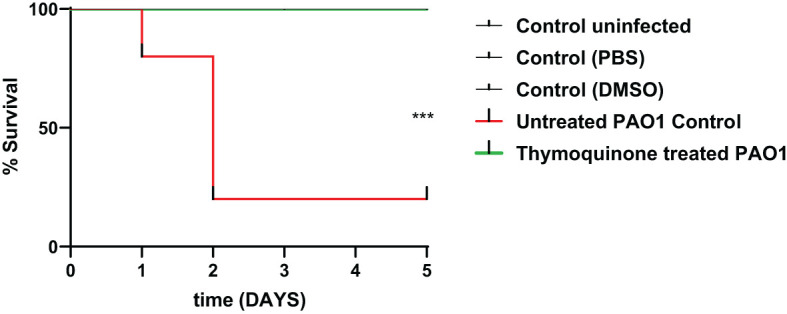
Thymoquinone reduced *P. aeruginosa* pathogenesis in the mouse infection model. Mouse survival was recorded using the Kaplan–Meier method, and the log-rank test for trend was employed to attest the statistical significance (*p* = 0.0005). Thymoquinone showed 100% protection as compared with 20% survival in mice injected with untreated bacteria. ***p < 0.001.

In order to test the protective activity of TQ against the pathogenic effects of *P. aeruginosa*, the mouse survival test was performed (**Figure 6**). In the negative control groups, namely, the uninfected mice group and the group injected with PBS or DMSO, the survival was 100%. However, the mice injected with untreated PAO1 (positive control group) began to die after 24 h and only 20% of mice survived at the end of the experiment. On the other hand, all mice injected with TQ-treated bacteria survived with no recorded deaths, which is similar to the observations in the negative control groups.

### Effect of thymoquinone on the expression of QS genes in *P. aeruginosa* PAO1

**Figure 7:**
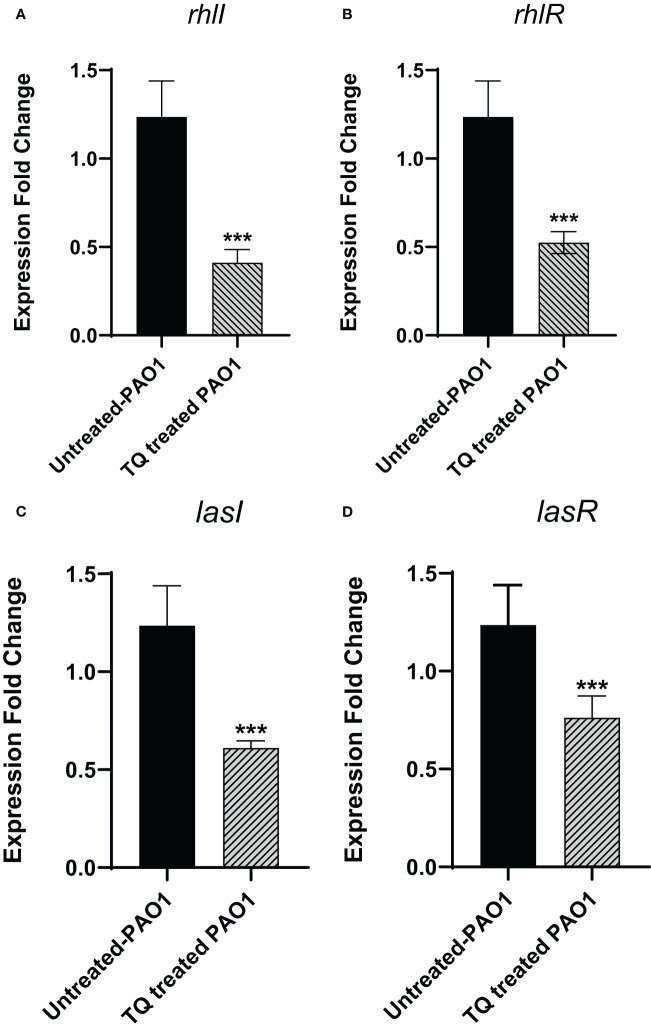
Thymoquinone significantly downregulated the expression of QS genes **(A)** *rhlI*, **(B)** *rhlR*. **(C)** *lasI*, and **(D)** *lasR*. The levels of expression were normalized to the housekeeping genes *ropD*. ***p < 0.001.

By investigation for its effect on the expression levels of QS genes *lasI*, *lasR*, *rhlI*, and *rhlR*, TQ significantly reduced the expression of all tested genes confirming the inhibitory effect against QS-controlled virulence. The relative expression of virulence factors regulating genes decreased approximately twofold (**Figure 7**).

### *In silico* multitarget docking analysis on QS receptors

**Figure 8:**
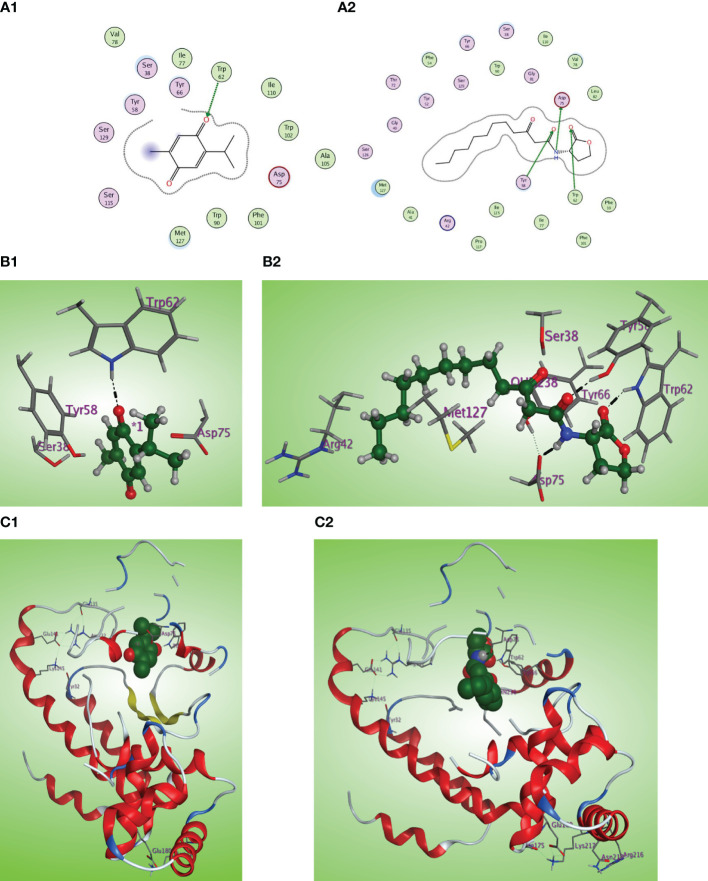
**(A1, 2)** Two-dimensional structural representation of the investigated thymoquinone and the reference ligand OHN in the active site of the QscR receptor (PDB: 3SZT). **(B1, 2)** Three-dimensional surface representation of the QscR receptor binding site with an overlay of thymoquinone and the reference ligand OHN (green ball and sticks). **(C1, 2)** Three-dimensional cartoon representations of the QscR receptor binding domain cocrystallized with thymoquinone and the reference ligand OHN (green space filling).

The *in-silico* study gives more insight into the antivirulence activity of TQ toward four *P. aeruginosa* targets QscR, LasR, RhlR, and PqsR quorum-sensing (QS) receptors. QscR responds to N-3-oxododecanoyl-L-homoserine lactone (3OC12-HSL). In this study, it was found that 3OC12-HSL makes many hydrogen bonds with QscR including Trp62, Tyr58, and Asp75 (Lintz et al., 2011). A validation approach was performed by redocking the reported ligand subtype of 3OC12-HSL (OHN) inside the LBD of QscR, and all the protruded hydrogen bonding interactions were confirmed (**Figures 8A2, B2, C2**). As the relative response of the wild-type QscR to different AHL is the same (Yao et al., 2006), we investigated the binding of TQ with its benzoquinone nucleus as an isostere to the lactone ring of ALHs and small methyl and isopropyl groups instead of the dodecyl extended flexible chain of ALHs. Thymoquinone revealed correct orientation inside the LBD with a respectable binding interaction (S = −5.8558 kcal/mol) and a prominent RMSD of 0.79815 from the normal ligand. The thymoquinone carbonyl group forms a characteristic hydrogen bonding with Trp62 at the LBD with a bond strength of −2.0 kcal/mol (**Figures 8A1, B1, C1**).

**Figure 9:**
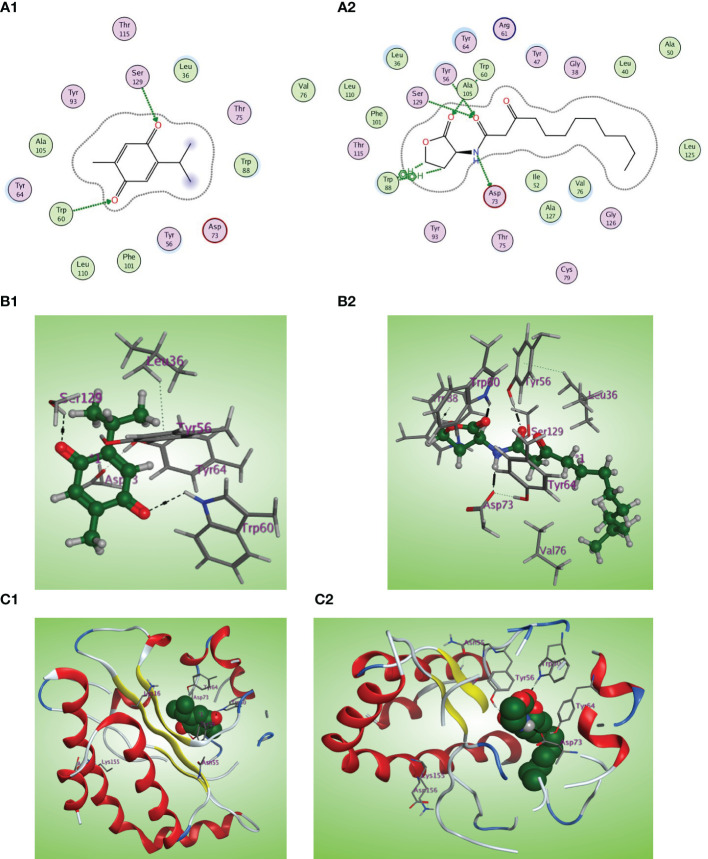
**(A1, 2)** Two-dimensional structural representation of the investigated thymoquinone and the reference ligand OHN in the active site of LasR receptor (PDB ID: 2UV0). **(B1, 2**) Three-dimensional surface representation of the LasR receptor binding site with an overlay of thymoquinone and the reference ligand OHN (green ball and sticks). **(C1, 2)** Three-dimensional cartoon representations of the LasR receptor binding domain cocrystallized with thymoquinone and the reference ligand OHN (green space filling).

The LasI synthase of *P. aeruginosa* produces the 3-oxo-C12-HSL that activates LasR (Smith and Iglewski, 2003). The natural ligand OHN exhibited important hydrogen bonding with the key Trp60, Tyr56, Ser129, and Asp73 residues in addition to hydrophobic interaction with the Trp88 residue (**Figures 9A2, B2, C2**). Thymoquinone demonstrated high binding affinity to the LasR LBD (S = −7.1001 kcal/mol) with prominent 2 hydrogen bonding acceptor effects with the same crucial Trp60 and Ser129 residues with binding energies of −0.8 and −1.0 kcal/mol, respectively (**Figures 9A1, B1, C1**).

**Figure 10:**
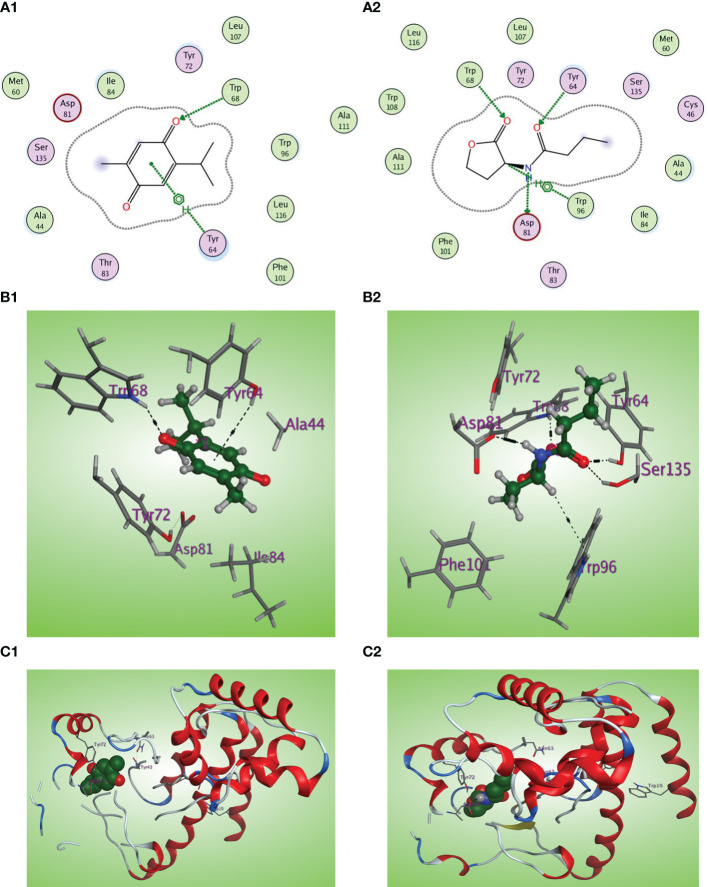
**(A1, 2)** Two-dimensional structural representation of the investigated thymoquinone and the reference ligand HL4 in the active site of RhlR receptor (PDB ID:7R3H). **(B1, 2)** Three-dimensional surface representation of the RhlR receptor binding site with an overlay of thymoquinone and the reference ligand HL4 (green ball and sticks). **(C1, 2)** Three-dimensional cartoon representations of the RhlR receptor binding domain co-crystallized with thymoquinone and the reference ligand HL4 (green space filling).

C4-HSL is the autoinducer of RhlR (Churchill and Chen, 2011). The natural ligand C4-HSL autoinducer subtype (HL4) could perform correct orientation and create characteristic hydrogen bonds with the side chains of Trp68, Tyr64, and Asp81 residues (**Figures 10A2, B2, C2**). Thymoquinone displays good affinity and correct orientation inside the LBD of the RhlR receptor with a binding energy of −5.5750 kcal/mol, and it created protruding hydrogen bonding with the key Trp62 reside with energy of −1.3 kcal/mol and a characteristic hydrophobic interaction with the critical Tyr64 residue with energy of −1.1 kcal/mol (**Figures 10A1, B1, C1**).

**Figure 11:**
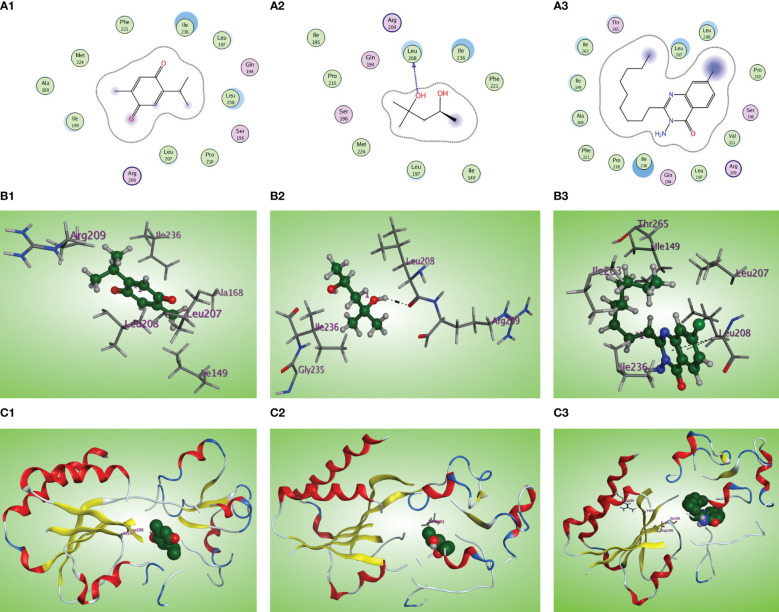
**(A1, 2, 3)** Two-dimensional structural representation of the investigated thymoquinone, the reference ligand MRD, and the 3-NH2-7Cl-C9-QZN inhibitor in the active site of the PqsR receptor (PDB ID: 4JVC). **(B1, 2, 3)** Three-dimensional surface representation of the PqsR receptor binding site with an overlay of thymoquinone, the reference ligand MRD, and the 3-NH2-7Cl-C9-QZN inhibitor (green ball and sticks). **(C1, 2, 3)** Three-dimensional cartoon representations of the PqsR receptor binding domain cocrystallized with thymoquinone, the reference ligand MRD, and the 3-NH2-7Cl-C9-QZN inhibitor (green space filling).

Concerning binding with the PqsR receptor, the PqsR inhibitor 3-amino-7-chlorinated QZN analogue, PqsR native ligand (MRD), and thymoquinone were docked inside the PqsR active site. All the docked compounds fit the same CBD of the PqsR receptor with different scoring functions, where the 3-NH2-7Cl-C9-QZN compound exerts the highest binding affinity (S = −5.4160 kcal/mol), whereas thymoquinone demonstrated higher PqsR binding affinity (S = −4.9740 kcal/mol) than the native ligand MRD (S = −4.6387 kcal/mol). Also, thymoquinone has high ligand exposure to the critical residue in the CBD such as Leu208 and Ile236 (**Figures 11A1, B1, C1**) like both the ligand MRD (**Figures 11A2, B2, C2**) and the inhibitor QZN analogue (**Figures 11A3, B3, C3**). The similar orientation of thymoquinone to the most potent QZN antagonist and the MRD natural ligand of PqsR, in addition to its high binding affinity at PqsR-CBD, makes it a potent inhibitor of the PqsR-QS receptor.

## Discussion

In this study, the inhibitory activity of thymoquinone (TQ) as a natural constituent against QS and its controlled virulence in *P. aeruginosa* was investigated. *P. aeruginosa* is a highly resistant healthcare-associated bacterium (Rossolini and Mantengoli, 2005). *P. aeruginosa* utilizes various virulence factors to elicit infections that may be chronic. Among these virulence factors, the pigment pyocyanin and the enzyme protease in addition to swarming motility and biofilm formation. Based on these facts, the approach of antivirulence therapy emerged to treat *P. aeruginosa* infections giving a promise to combat the problem of antibiotic resistance (Hentzer et al., 2003; Bjarnsholt et al., 2010; Garcia-Contreras, 2016).

First, TQ MIC was determined to select the subinhibitory concentration used in our study. Utilizing anti-QS and antivirulence agents at sub-MIC prevents undue stress on bacteria, preventing the development of resistance (Hentzer et al., 2003; Jakobsen et al., 2013). TQ inhibited the growth of *P. aeruginosa* PAO1 strain at 500 μg/ml; therefore, ¼ MIC or 125 μg/ml was used to test the potential inhibitory activity of TQ on virulence. Furthermore, the effect of this concentration on bacterial growth was assessed and it was found that it did not affect the growth.

The role of QS in the formation of bacterial biofilms represents a crucial aspect of microbial behavior (Li and Tian, 2012). As bacteria reach a quorum, they initiate the production of extracellular polymeric substances (EPS), which form the matrix that holds the biofilm together (Lopez et al., 2010; Delcaru et al., 2016). The ability of bacteria to form biofilms through QS is not only a survival strategy but also a significant factor in their virulence and contributes to the chronic nature of biofilm-associated infections (Kirisits and Parsek, 2006; Lila et al., 2023). Remarkably, our study revealed that subinhibitory concentrations of TQ had a significant inhibitory effect on biofilm formation, reducing it by over 60%. Bearing in mind that swarming motility enables the movement of *P. aeruginosa* and is associated with the formation of biofilms (Coleman et al., 2021). Additionally, swarmer cells demonstrate heightened antibiotic resistance and an increased production of virulence factors (Floyd et al., 2016). Significant reduction of swarming motility was found in this study by TQ at sub-MIC. This finding confirms the potential of TQ as a promising antibiofilm agent.

QS systems exhibit varied functions in regulating the expression of numerous virulence factors. Consequently, they play a crucial role in governing bacterial pathogenesis and their capacity to initiate infections (Venturi, 2006; Papenfort and Bassler, 2016). Pyocyanin was found to enhance the ability of *P. aeruginosa* to penetrate the cell membranes of the host tissues. Moreover, it damages the cells by interference with its functions (Hall et al., 2016). The virulent enzyme protease exerts various roles associated with the pathogenesis of *P. aeruginosa*. It can decompose the host lung elastin damaging the lung particularly in patients suffering from cystic fibrosis. Moreover, protease can decompose immunoglobulins and fibrin in addition to epithelial tight junctions (Kipnis et al., 2006). In the current study, TQ significantly decreased the secretion of pyocyanin pigment and the production of protease that indicate a potential antivirulence activity of TQ.

*P. aeruginosa* utilizes two main LuxI/LuxR QS systems to harmonize the pathogenicity. The LuxI systems are LasI and RhlI that are responsible for the secretion of the autoinducers that bind to the LuxR homologs, namely, LasR and RhlR receptors (Fu et al., 2015; Hema et al., 2017). In our study, TQ could remarkably downregulate the QS encoding genes *lasI*, *lasR*, *rhlI*, and *rhlR*, which suggests a potential anti-QS activity of TQ. It is worthy to declare that *P. aeruginosa* acquires another orphan Lux-type QS system QscR which senses the LasI autoinducers (Papenfort and Bassler, 2016). Furthermore, *P. aeruginosa* employs its non-Lux type QS (PQS) system which is expressed by the operon *pqsA-E* (Juhas et al., 2005; Venturi, 2006). A detailed docking analysis of TQ was carried out on the four QS receptors, revealing a notable affinity of TQ to bind and disrupt these receptors. This implies a potential inhibitory activity against quorum sensing.

To further confirm the antivirulence activity of TQ the *in vivo* protection assay was conducted. By detection of the mouse survival after injection of TQ-treated and control untreated bacteria. In harmony with *in vitro* phenotypic and genotypic results and virtual docking study, TQ increased the survival of mice from only 20% in the group infected with untreated bacteria to 100% in mice infected with TQ-treated bacteria.

TQ has the advantage of being safe and natural. It is present in *Nigella sativa* (black cumin) seeds as a major constituent with a content of 30% to 48% (Ahmad et al., 2016). It exhibits antibacterial and antifungal activities (Halawani, 2009; Ragheb et al., 2009). We demonstrated the potential anti-QS and antivirulence activities of TQ at a concentration of 125 μg/ml; it is noteworthy that this concentration is deemed safe. This safety profile is supported by previous findings indicating that a dose equivalent to 200 mg/adult/day was well-tolerated in a phase 1 clinical trial targeting sleep disorders and stress using a formulation comprising black cumin oil with 5% (50 mg/ml) TQ utilized (Thomas et al., 2022).

The use of natural compounds as antivirulence and anti-QS agents was previously reported. Methyl gallate showed a strong inhibition of QS in *P. aeruginosa* strains PAO1 and PA14 at ¼ MICs without affecting bacterial viability (Naga et al., 2023). Moreover, flavanones, naringenin, eriodictyol, and taxifolin extracted from *Combretum albiflorum* interfered with pyocyanin production and elastase activity in *P. aeruginosa* without impacting bacterial growth. Taxifolin and naringenin could downregulate QS-controlled genes *lasI*, *lasR*, *rhlI*, and *rhlR* in *P. aeruginosa* PAO1 (Vandeputte et al., 2011). Another natural agent is quercetin that reduced the QS-controlled biofilm formation, pyocyanin, protease, elastase, and motility by competition for the lasR receptor (Gopu et al., 2015). At the molecular level, quercetin decreased the expression of the genes *lasI*, *lasR*, *rhlI*, and *rhlR* (Ouyang et al., 2016). In our previous study, the natural agent sotolon present in the seeds of Fenugreek could inhibit virulence and QS in *P. aeruginosa*. Significant inhibition of biofilm, protease, elastase, and pyocyanin was achieved with sotolon. Also, it reduced the expression of *lasI*, *lasR*, *rhlI*, and *rhlR* and protected mice against the pathogenesis of *P. aeruginosa* (Aldawsari et al., 2021). Here, TQ is a promising antivirulence candidate and could interfere with QS systems at relatively low concentration 125 µg/mL (sub-MIC) and could be used in combination with traditional antibiotics in treatment of pseudomonal infections and other serious infections.

## Conclusion

Thymoquinone (TQ) emerges as a safe natural compound suitable for the treatment of *P. aeruginosa* infections. It could inhibit biofilm formation, reduce production of virulence factors as proteases and pyocyanin and attenuate pathogenicity through its QS inhibitory activity. The present phenotypic, genotypic, *in silico*, and *in vivo* investigations show the prospective antibiofilm and antivirulence activities of TQ. Moreover, the benzoquinone ring may be a good pharmacophore for synthesis of potent anti-QS and antivirulence compounds for treating bacterial infections.

## Data availability statement

The raw data supporting the conclusions of this article will be made available by the authors, without undue reservation.

## Ethics statement

Ethical approval for the *in vivo* experiment was obtained from Zagazig University Institutional Animal Care and Use Committee with approval number of (ZU-IACUC/3/F/392/2022). The study was conducted in accordance with the local legislation and institutional requirements.

## Author contributions

MA-R: Writing – original draft, Visualization, Methodology, Investigation. HZA: Writing – original draft, Resources, Methodology, Funding acquisition, Data curation. NA: Writing – original draft, Resources, Funding acquisition, Formal analysis, Data curation. WA: Writing – original draft, Software, Data curation. TI: Writing – original draft, Software, Resources, Investigation. HAA: Writing – review & editing, Writing – original draft, Validation, Supervision, Conceptualization. IS: Writing – original draft, Visualization, Methodology, Software, Investigation, Formal analysis. WH: Writing – review & editing, Writing – original draft, Validation, Supervision, Project administration, Conceptualization. SN: Writing – original draft, Methodology, Investigation, Formal analysis, Data curation.
